# 3-(Adamantan-1-yl)-4-benzyl-1*H*-1,2,4-triazole-5(4*H*)-thione

**DOI:** 10.1107/S1600536814013257

**Published:** 2014-06-14

**Authors:** Fatmah A. M. Al-Omary, Hazem A. Ghabbour, Ali A. El-Emam, C. S. Chidan Kumar, Hoong-Kun Fun

**Affiliations:** a Department of Pharmaceutical Chemistry, College of Pharmacy, King Saud University, PO Box 2457, Riaydh 11451, Saudi Arabia; b King Abdullah Institute for Nanotechnology (KAIN), King Saud University, Riyadh 11451, Saudi Arabia; c X-ray Crystallography Unit, School of Physics, Universiti Sains Malaysia, 11800 USM, Penang, Malaysia

## Abstract

The title compound, C_19_H_23_N_3_S, is a functionalized triazoline-3-thione derivative. The benzyl ring is almost normal to the planar 1,2,4-triazole ring (r.m.s. deviation = 0.007 Å) with a dihedral angle of 86.90 (7)°. In the crystal, molecules are linked by pairs of N—H⋯S hydrogen bonds, forming inversion dimers that enclose *R*_2_^2^(8) loops. The crystal packing is further stabilized by weak C—H⋯π inter­actions that link adjacent dimeric units into supra­molecular chains extending along the *a*-axis direction.

## Related literature   

For the biological activity of adamantane derivatives, see: Lorenzo *et al.* (2008 ▶); Al-Deeb *et al.* (2006 ▶); Wang *et al.* (2013 ▶); El-Emam *et al.* (2004 ▶); Kadi *et al.* (2010 ▶); Balzarini *et al.* (2009 ▶); Protopopova *et al.* (2005 ▶); Vernier *et al.* (1969 ▶). For related adamantyl-1,2,4-triazole structures, see: El-Emam *et al.* (2012 ▶), Al-Tamimi *et al.* (2013 ▶). For the synthesis of the title compound, see El-Emam & Ibrahim (1991 ▶). For hydrogen-bond motifs, see: Bernstein *et al.* (1995 ▶).
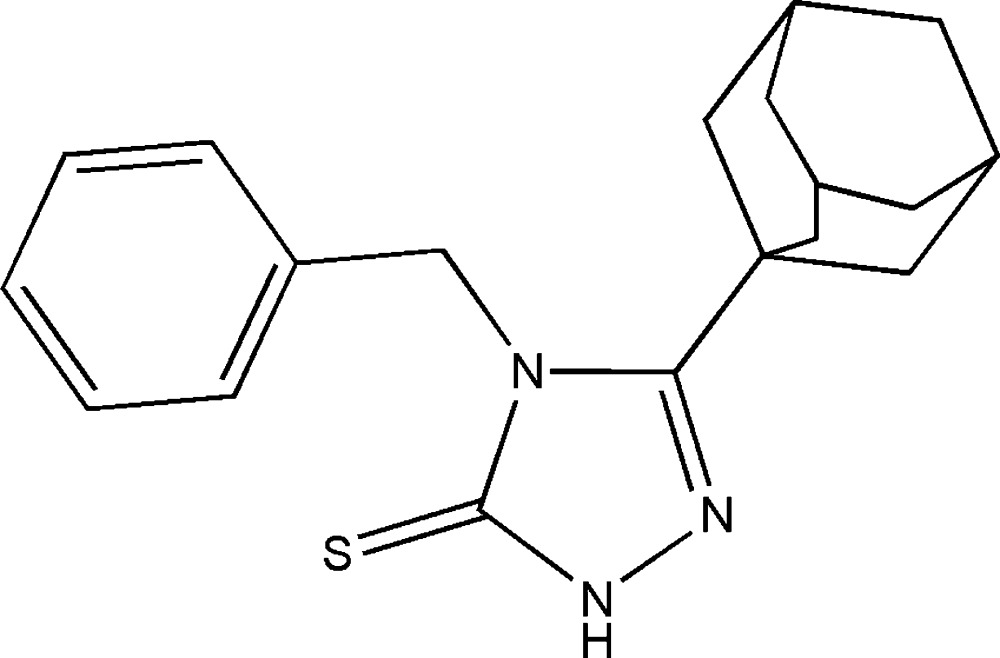


## Experimental   

### 

#### Crystal data  

C_19_H_23_N_3_S
*M* *_r_* = 325.46
Triclinic, 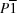

*a* = 7.6407 (4) Å
*b* = 10.5150 (5) Å
*c* = 12.3434 (5) Å
α = 67.1806 (13)°
β = 72.9688 (13)°
γ = 70.0695 (14)°
*V* = 844.42 (7) Å^3^
*Z* = 2
Mo *K*α radiation
μ = 0.20 mm^−1^
*T* = 293 K
0.60 × 0.48 × 0.34 mm

#### Data collection  

Bruker APEXII CCD diffractometer
Absorption correction: multi-scan (*SADABS*; Bruker, 2009 ▶) *T*_min_ = 0.891, *T*_max_ = 0.937
43581 measured reflections
5166 independent reflections
4651 reflections with *I* > 2σ(*I*)
*R*_int_ = 0.029

#### Refinement  

*R*[*F* ^2^ > 2σ(*F* ^2^)] = 0.046
*wR*(*F* ^2^) = 0.127
*S* = 1.08
5166 reflections
212 parameters
H atoms treated by a mixture of independent and constrained refinement
Δρ_max_ = 0.31 e Å^−3^
Δρ_min_ = −0.56 e Å^−3^

### 

Data collection: *APEX2* (Bruker, 2009 ▶); cell refinement: *SAINT* (Bruker, 2009 ▶); data reduction: *SAINT*; program(s) used to solve structure: *SHELXS97* (Sheldrick, 2008 ▶); program(s) used to refine structure: *SHELXL97* (Sheldrick, 2008 ▶); molecular graphics: *SHELXTL* (Sheldrick, 2008 ▶); software used to prepare material for publication: *SHELXTL* and *PLATON* (Spek, 2009 ▶).

## Supplementary Material

Crystal structure: contains datablock(s) global, I. DOI: 10.1107/S1600536814013257/sj5408sup1.cif

Structure factors: contains datablock(s) I. DOI: 10.1107/S1600536814013257/sj5408Isup2.hkl

Supporting information file. DOI: 10.1107/S1600536814013257/sj5408Isup3.cml

CCDC reference: 1007119

Additional supporting information:  crystallographic information; 3D view; checkCIF report

## Figures and Tables

**Table 1:** Hydrogen-bond geometry (Å, °) *Cg*1 is the centroid of the N1–N3/C8/C9 triazole ring.

*D*—H⋯*A*	*D*—H	H⋯*A*	*D*⋯*A*	*D*—H⋯*A*
N2—H1*N*2⋯S1^i^	0.85 (2)	2.44 (2)	3.2753 (11)	169.1 (18)
C19—H19*B*⋯*Cg*1^ii^	0.97	2.85	3.7885 (17)	141

Symmetry codes: (i) 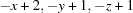; (ii) 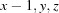.

**Fig. 1.**
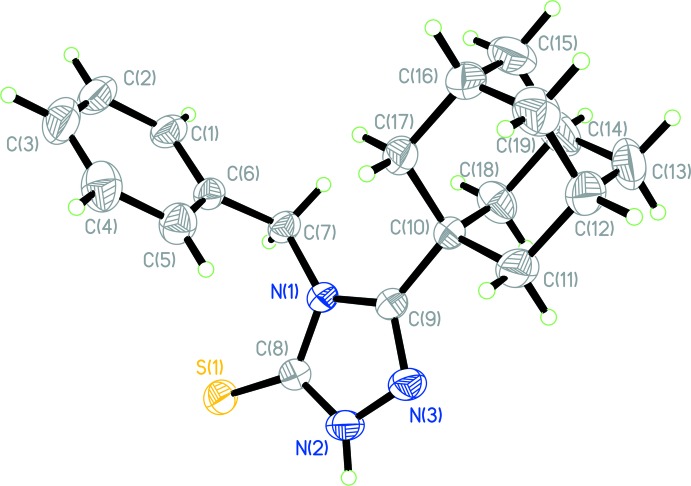
The molecular structure of the title compound with atom labels and 50% probability displacement ellipsoids.

**Fig. 2.**
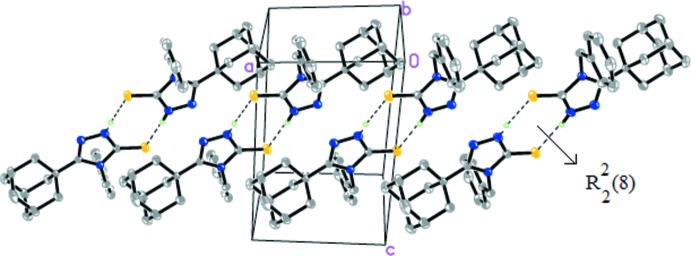
Crystal packing of the title compound, showing the hydrogen bonding interactions as dashed lines. H-atoms not involved in the hydrogen bonding are omitted for clarity.

## supplementary crystallographic information

### S1. Comment

Adamantane derivatives have long been known for their diverse biological activities (Lorenzo *et al.*, 2008; Al-Deeb *et al.*, 2006; Wang *et al.*, 2013). These also include antiviral activity against influenza (Vernier *et al.*, 1969) and HIV viruses (El-Emam *et al.*, 2004; Balzarini *et al.*, 2009). In addition, adamantane derivative were recently reported to exhibit marked antibacterial activity (Kadi *et al*., 2010; Protopopova *et al.*, 2005). In an earlier publication, we reported the synthesis and potent anti-inflammatory of a series of 5-(1-adamantyl)-4-substituted-4H-1,2,4-triazole-3-thiols and related derivatives including the title compound (El-Emam & Ibrahim, 1991).

In the title compound (Fig. 1), the 1,2,4-triazole (N1—N3/C8/C9) ring is nearly planar with a maximum deviation of -0.007 (1) Å at atom N2. The central 1,2,4-triazole ring forms dihedral angles of 86.90 (7)° and 69 (4)° with the adjacent phenyl (C1–C6) and adamantyl (C10–C19) substituents attached at the 4- and 5-positions, respectively. The attached phenyl ring is almost perpendicular to the plane of the triazole which is evident from the C9–N1–C7–C6 torsion angle of -95.63 (12)°. In the crystal packing (Fig. 2), centrosymmetric dimeric aggregates are formed by pairs of N2—H1N2···S1 hydrogen bonds resulting in an *R*_2_^2^(8) ring motif (Bernstein *et al.*, 1995). These are connected into supramolecular chains extending along the *a* axis direction *via* weak intermolecular C–H···π(triazole) interactions (Table 1).

### S2. Experimental

A mixture of adamantane-1-carbohydrazide (1.94 g, 0.01 mol), benzyl isothiocyanate (1.49 g, 0.01 mol), in ethanol (10 ml) was heated under reflux with stirring for one hour and the solvent was distilled off *in vacuo*. Aqueous sodium hydroxide solution (10%, 15 ml) was added to the residue and the mixture was heated under reflux for 2 h. then filtered hot. On cooling, the mixture was acidified with hydrochloric acid and the precipitated crude product was filtered, washed with water, dried and crystallized from aqueous ethanol to yield 2.93 g (90%) of the title compound (C_19_H_23_N_3_S) as colorless crystals. M·P.: 241–243 °C.

^1^H NMR (CDCl_3_, 700.17 MHz): δ 1.64–1.69 (m, 6H, Adamantane-H), 1.90 (s, 6H, Adamantane-H), 2.20 (s, 3H, Adamantane-H), 5.53 (s, 2H, CH_2_), 7.04–7.63 (s, 5H, Ar—H), 11.55 (br. s, 1H, NH). ^13^C NMR (CDCl_3_, 176.08 MHz): δ 28.51, 35.66, 36.86, 39.08 (Adamantane-C), 63.56 (CH_2_), 121.25, 123.0, 124.27, 130.54 (Ar—C), 154.06 (C=N), 164.41 (C=S).

### S3. Refinement

The nitrogen-bound H-atom was located in a difference Fourier map and was refined freely. Other H atoms were positioned geometrically (C=H 0.93–0.98 Å) and refined using a riding model with *U*_iso_(H) = 1.2 *U*_eq_(C) or 1.5 *U*_eq_(C) for methyl H atoms. A rotating group model was used for the methyl group.

### Crystal data

**Table:**

C_19_H_23_N_3_S	*Z* = 2
*M**_r_* = 325.46	*F*(000) = 348
Triclinic, *P*1	*D*_x_ = 1.280 Mg m^−^^3^
Hall symbol: -P 1	Mo *K*α radiation, λ = 0.71073 Å
*a* = 7.6407 (4) Å	Cell parameters from 9608 reflections
*b* = 10.5150 (5) Å	θ = 2.9–30.6°
*c* = 12.3434 (5) Å	µ = 0.20 mm^−^^1^
α = 67.1806 (13)°	*T* = 293 K
β = 72.9688 (13)°	Block, colourless
γ = 70.0695 (14)°	0.60 × 0.48 × 0.34 mm
*V* = 844.42 (7) Å^3^	

### Data collection

**Table:**

Bruker APEXII CCD diffractometer	5166 independent reflections
Radiation source: fine-focus sealed tube	4651 reflections with *I* > 2σ(*I*)
Graphite monochromator	*R*_int_ = 0.029
φ and ω scans	θ_max_ = 30.6°, θ_min_ = 2.2°
Absorption correction: multi-scan (*SADABS*; Bruker, 2009)	*h* = −10→10
*T*_min_ = 0.891, *T*_max_ = 0.937	*k* = −15→15
43581 measured reflections	*l* = −17→17

### Refinement

**Table:**

Refinement on *F*^2^	Primary atom site location: structure-invariant direct methods
Least-squares matrix: full	Secondary atom site location: difference Fourier map
*R*[*F*^2^ > 2σ(*F*^2^)] = 0.046	Hydrogen site location: inferred from neighbouring sites
*wR*(*F*^2^) = 0.127	H atoms treated by a mixture of independent and constrained refinement
*S* = 1.08	*w* = 1/[σ^2^(*F*_o_^2^) + (0.0747*P*)^2^ + 0.1424*P*] where *P* = (*F*_o_^2^ + 2*F*_c_^2^)/3
5166 reflections	(Δ/σ)_max_ = 0.001
212 parameters	Δρ_max_ = 0.31 e Å^−^^3^
0 restraints	Δρ_min_ = −0.56 e Å^−^^3^

### Special details

**Table:**

Geometry. All e.s.d.'s (except the e.s.d. in the dihedral angle between two l.s. planes) are estimated using the full covariance matrix. The cell e.s.d.'s are taken into account individually in the estimation of e.s.d.'s in distances, angles and torsion angles; correlations between e.s.d.'s in cell parameters are only used when they are defined by crystal symmetry. An approximate (isotropic) treatment of cell e.s.d.'s is used for estimating e.s.d.'s involving l.s. planes.
Refinement. Refinement of *F*^2^ against ALL reflections. The weighted *R*-factor *wR* and goodness of fit *S* are based on *F*^2^, conventional *R*-factors *R* are based on *F*, with *F* set to zero for negative *F*^2^. The threshold expression of *F*^2^ > σ(*F*^2^) is used only for calculating *R*-factors(gt) *etc*. and is not relevant to the choice of reflections for refinement. *R*-factors based on *F*^2^ are statistically about twice as large as those based on *F*, and *R*- factors based on ALL data will be even larger.

### Fractional atomic coordinates and isotropic or equivalent isotropic displacement parameters (Å^2^)

**Table:**

	*x*	*y*	*z*	*U*_iso_*/*U*_eq_	
S1	1.05370 (4)	0.44991 (3)	0.32578 (3)	0.03965 (10)	
N1	0.70863 (12)	0.63160 (9)	0.28216 (7)	0.02792 (16)	
N2	0.74865 (14)	0.58334 (11)	0.45903 (8)	0.0360 (2)	
N3	0.57266 (14)	0.67716 (11)	0.45231 (8)	0.0359 (2)	
C1	0.75626 (15)	0.49115 (13)	0.03402 (10)	0.0363 (2)	
H1A	0.8142	0.5555	−0.0302	0.044*	
C2	0.71844 (19)	0.38114 (15)	0.01658 (13)	0.0471 (3)	
H2A	0.7512	0.3719	−0.0593	0.057*	
C3	0.6325 (2)	0.28550 (15)	0.11137 (15)	0.0522 (3)	
H3A	0.6073	0.2118	0.0995	0.063*	
C4	0.5840 (2)	0.29922 (14)	0.22381 (14)	0.0497 (3)	
H4A	0.5253	0.2349	0.2876	0.060*	
C5	0.62220 (17)	0.40876 (12)	0.24249 (10)	0.0390 (2)	
H5A	0.5903	0.4169	0.3187	0.047*	
C6	0.70774 (13)	0.50549 (10)	0.14754 (8)	0.02895 (19)	
C7	0.75127 (15)	0.62875 (11)	0.15983 (8)	0.03048 (19)	
H7A	0.8846	0.6242	0.1282	0.037*	
H7B	0.6793	0.7175	0.1113	0.037*	
C8	0.83679 (14)	0.55430 (10)	0.35740 (9)	0.03030 (19)	
C9	0.54907 (13)	0.70511 (10)	0.34398 (8)	0.02803 (18)	
C10	0.37568 (13)	0.81094 (10)	0.29687 (8)	0.02757 (18)	
C11	0.22454 (18)	0.84649 (16)	0.40233 (11)	0.0460 (3)	
H11A	0.1894	0.7604	0.4584	0.055*	
H11B	0.2761	0.8830	0.4439	0.055*	
C12	0.04846 (19)	0.95844 (17)	0.35680 (13)	0.0513 (3)	
H12A	−0.0461	0.9806	0.4247	0.062*	
C13	0.1044 (2)	1.09463 (16)	0.26935 (19)	0.0642 (4)	
H13A	0.1566	1.1326	0.3094	0.077*	
H13B	−0.0065	1.1663	0.2411	0.077*	
C14	0.2523 (2)	1.05937 (13)	0.16312 (15)	0.0553 (4)	
H14A	0.2873	1.1465	0.1064	0.066*	
C15	0.1686 (2)	0.99973 (17)	0.10091 (13)	0.0546 (3)	
H15A	0.0585	1.0705	0.0709	0.065*	
H15B	0.2614	0.9773	0.0336	0.065*	
C16	0.11210 (18)	0.86562 (14)	0.18917 (12)	0.0436 (3)	
H16A	0.0581	0.8279	0.1488	0.052*	
C17	0.28778 (16)	0.75284 (12)	0.23410 (11)	0.0380 (2)	
H17A	0.3800	0.7290	0.1672	0.046*	
H17B	0.2524	0.6667	0.2897	0.046*	
C18	0.42886 (18)	0.94874 (12)	0.20848 (12)	0.0428 (3)	
H18A	0.4818	0.9870	0.2479	0.051*	
H18B	0.5239	0.9277	0.1415	0.051*	
C19	−0.03504 (18)	0.90065 (16)	0.29388 (14)	0.0499 (3)	
H19A	−0.1464	0.9712	0.2654	0.060*	
H19B	−0.0728	0.8153	0.3493	0.060*	
H1N2	0.798 (3)	0.5628 (19)	0.5188 (16)	0.056 (5)*	

### Atomic displacement parameters (Å^2^)

**Table:**

	*U*^11^	*U*^22^	*U*^33^	*U*^12^	*U*^13^	*U*^23^
S1	0.03414 (15)	0.04240 (16)	0.04517 (17)	0.00090 (11)	−0.01292 (11)	−0.02211 (12)
N1	0.0301 (4)	0.0295 (4)	0.0249 (3)	−0.0049 (3)	−0.0058 (3)	−0.0114 (3)
N2	0.0363 (4)	0.0423 (5)	0.0276 (4)	−0.0032 (4)	−0.0107 (3)	−0.0118 (3)
N3	0.0351 (4)	0.0435 (5)	0.0264 (4)	−0.0027 (4)	−0.0068 (3)	−0.0140 (3)
C1	0.0341 (5)	0.0463 (6)	0.0330 (5)	−0.0067 (4)	−0.0059 (4)	−0.0208 (4)
C2	0.0439 (6)	0.0582 (7)	0.0537 (7)	−0.0057 (5)	−0.0127 (5)	−0.0368 (6)
C3	0.0468 (7)	0.0482 (7)	0.0780 (9)	−0.0082 (5)	−0.0187 (6)	−0.0355 (7)
C4	0.0485 (7)	0.0399 (6)	0.0619 (8)	−0.0167 (5)	−0.0066 (6)	−0.0158 (5)
C5	0.0414 (6)	0.0381 (5)	0.0367 (5)	−0.0109 (4)	−0.0020 (4)	−0.0144 (4)
C6	0.0261 (4)	0.0323 (4)	0.0295 (4)	−0.0029 (3)	−0.0052 (3)	−0.0149 (3)
C7	0.0348 (5)	0.0337 (5)	0.0238 (4)	−0.0092 (4)	−0.0022 (3)	−0.0122 (3)
C8	0.0327 (4)	0.0295 (4)	0.0302 (4)	−0.0067 (3)	−0.0087 (3)	−0.0103 (3)
C9	0.0301 (4)	0.0295 (4)	0.0242 (4)	−0.0063 (3)	−0.0044 (3)	−0.0101 (3)
C10	0.0292 (4)	0.0276 (4)	0.0256 (4)	−0.0053 (3)	−0.0050 (3)	−0.0101 (3)
C11	0.0372 (5)	0.0618 (7)	0.0336 (5)	0.0026 (5)	−0.0053 (4)	−0.0240 (5)
C12	0.0377 (6)	0.0647 (8)	0.0516 (7)	0.0076 (5)	−0.0096 (5)	−0.0357 (6)
C13	0.0572 (8)	0.0428 (7)	0.1067 (13)	0.0107 (6)	−0.0372 (9)	−0.0429 (8)
C14	0.0536 (7)	0.0294 (5)	0.0729 (9)	−0.0095 (5)	−0.0244 (7)	0.0022 (5)
C15	0.0523 (7)	0.0577 (8)	0.0418 (6)	−0.0021 (6)	−0.0200 (6)	−0.0058 (6)
C16	0.0399 (6)	0.0473 (6)	0.0527 (7)	−0.0034 (5)	−0.0192 (5)	−0.0243 (5)
C17	0.0377 (5)	0.0351 (5)	0.0480 (6)	−0.0052 (4)	−0.0136 (4)	−0.0199 (4)
C18	0.0402 (6)	0.0310 (5)	0.0536 (7)	−0.0126 (4)	−0.0123 (5)	−0.0043 (4)
C19	0.0324 (5)	0.0551 (7)	0.0610 (8)	−0.0069 (5)	−0.0090 (5)	−0.0207 (6)

### Geometric parameters (Å, º)

**Table:**

S1—C8	1.6784 (10)	C10—C17	1.5415 (14)
N1—C8	1.3735 (12)	C11—C12	1.5368 (18)
N1—C9	1.3915 (12)	C11—H11A	0.9700
N1—C7	1.4586 (12)	C11—H11B	0.9700
N2—C8	1.3351 (13)	C12—C19	1.517 (2)
N2—N3	1.3732 (13)	C12—C13	1.532 (3)
N2—H1N2	0.846 (19)	C12—H12A	0.9800
N3—C9	1.3065 (12)	C13—C14	1.535 (3)
C1—C2	1.3876 (16)	C13—H13A	0.9700
C1—C6	1.3946 (13)	C13—H13B	0.9700
C1—H1A	0.9300	C14—C15	1.525 (2)
C2—C3	1.378 (2)	C14—C18	1.5344 (18)
C2—H2A	0.9300	C14—H14A	0.9800
C3—C4	1.379 (2)	C15—C16	1.520 (2)
C3—H3A	0.9300	C15—H15A	0.9700
C4—C5	1.3929 (17)	C15—H15B	0.9700
C4—H4A	0.9300	C16—C19	1.5185 (19)
C5—C6	1.3840 (15)	C16—C17	1.5351 (16)
C5—H5A	0.9300	C16—H16A	0.9800
C6—C7	1.5131 (13)	C17—H17A	0.9700
C7—H7A	0.9700	C17—H17B	0.9700
C7—H7B	0.9700	C18—H18A	0.9700
C9—C10	1.5086 (13)	C18—H18B	0.9700
C10—C11	1.5396 (14)	C19—H19A	0.9700
C10—C18	1.5396 (14)	C19—H19B	0.9700
			
C8—N1—C9	108.06 (8)	C19—C12—C13	109.53 (12)
C8—N1—C7	121.22 (8)	C19—C12—C11	109.84 (11)
C9—N1—C7	130.71 (8)	C13—C12—C11	109.41 (12)
C8—N2—N3	113.41 (9)	C19—C12—H12A	109.3
C8—N2—H1N2	126.3 (12)	C13—C12—H12A	109.3
N3—N2—H1N2	119.1 (12)	C11—C12—H12A	109.3
C9—N3—N2	104.79 (8)	C12—C13—C14	109.13 (10)
C2—C1—C6	120.22 (11)	C12—C13—H13A	109.9
C2—C1—H1A	119.9	C14—C13—H13A	109.9
C6—C1—H1A	119.9	C12—C13—H13B	109.9
C3—C2—C1	120.15 (11)	C14—C13—H13B	109.9
C3—C2—H2A	119.9	H13A—C13—H13B	108.3
C1—C2—H2A	119.9	C15—C14—C18	109.88 (12)
C2—C3—C4	119.91 (11)	C15—C14—C13	109.38 (12)
C2—C3—H3A	120.0	C18—C14—C13	109.37 (13)
C4—C3—H3A	120.0	C15—C14—H14A	109.4
C3—C4—C5	120.43 (12)	C18—C14—H14A	109.4
C3—C4—H4A	119.8	C13—C14—H14A	109.4
C5—C4—H4A	119.8	C16—C15—C14	109.44 (11)
C6—C5—C4	119.92 (11)	C16—C15—H15A	109.8
C6—C5—H5A	120.0	C14—C15—H15A	109.8
C4—C5—H5A	120.0	C16—C15—H15B	109.8
C5—C6—C1	119.36 (10)	C14—C15—H15B	109.8
C5—C6—C7	123.23 (9)	H15A—C15—H15B	108.2
C1—C6—C7	117.40 (9)	C19—C16—C15	109.94 (11)
N1—C7—C6	114.42 (8)	C19—C16—C17	109.96 (11)
N1—C7—H7A	108.7	C15—C16—C17	109.53 (10)
C6—C7—H7A	108.7	C19—C16—H16A	109.1
N1—C7—H7B	108.7	C15—C16—H16A	109.1
C6—C7—H7B	108.7	C17—C16—H16A	109.1
H7A—C7—H7B	107.6	C16—C17—C10	109.85 (9)
N2—C8—N1	103.79 (9)	C16—C17—H17A	109.7
N2—C8—S1	129.01 (8)	C10—C17—H17A	109.7
N1—C8—S1	127.19 (8)	C16—C17—H17B	109.7
N3—C9—N1	109.93 (9)	C10—C17—H17B	109.7
N3—C9—C10	122.14 (9)	H17A—C17—H17B	108.2
N1—C9—C10	127.80 (8)	C14—C18—C10	109.81 (9)
C9—C10—C11	108.87 (8)	C14—C18—H18A	109.7
C9—C10—C18	109.22 (8)	C10—C18—H18A	109.7
C11—C10—C18	108.93 (10)	C14—C18—H18B	109.7
C9—C10—C17	112.77 (8)	C10—C18—H18B	109.7
C11—C10—C17	107.69 (9)	H18A—C18—H18B	108.2
C18—C10—C17	109.28 (9)	C12—C19—C16	109.24 (10)
C12—C11—C10	110.21 (10)	C12—C19—H19A	109.8
C12—C11—H11A	109.6	C16—C19—H19A	109.8
C10—C11—H11A	109.6	C12—C19—H19B	109.8
C12—C11—H11B	109.6	C16—C19—H19B	109.8
C10—C11—H11B	109.6	H19A—C19—H19B	108.3
H11A—C11—H11B	108.1		
			
C8—N2—N3—C9	1.25 (13)	N3—C9—C10—C17	−132.65 (10)
C6—C1—C2—C3	0.06 (19)	N1—C9—C10—C17	51.96 (13)
C1—C2—C3—C4	0.0 (2)	C9—C10—C11—C12	177.91 (10)
C2—C3—C4—C5	−0.4 (2)	C18—C10—C11—C12	58.90 (14)
C3—C4—C5—C6	0.7 (2)	C17—C10—C11—C12	−59.52 (13)
C4—C5—C6—C1	−0.56 (17)	C10—C11—C12—C19	60.43 (15)
C4—C5—C6—C7	179.01 (11)	C10—C11—C12—C13	−59.83 (15)
C2—C1—C6—C5	0.21 (16)	C19—C12—C13—C14	−60.12 (15)
C2—C1—C6—C7	−179.39 (10)	C11—C12—C13—C14	60.33 (15)
C8—N1—C7—C6	85.08 (11)	C12—C13—C14—C15	59.48 (15)
C9—N1—C7—C6	−95.63 (12)	C12—C13—C14—C18	−60.91 (15)
C5—C6—C7—N1	4.38 (14)	C18—C14—C15—C16	60.57 (16)
C1—C6—C7—N1	−176.04 (9)	C13—C14—C15—C16	−59.51 (15)
N3—N2—C8—N1	−1.21 (12)	C14—C15—C16—C19	60.18 (14)
N3—N2—C8—S1	178.19 (8)	C14—C15—C16—C17	−60.75 (14)
C9—N1—C8—N2	0.70 (11)	C19—C16—C17—C10	−60.82 (13)
C7—N1—C8—N2	−179.87 (9)	C15—C16—C17—C10	60.09 (13)
C9—N1—C8—S1	−178.72 (7)	C9—C10—C17—C16	179.74 (9)
C7—N1—C8—S1	0.71 (14)	C11—C10—C17—C16	59.61 (12)
N2—N3—C9—N1	−0.74 (11)	C18—C10—C17—C16	−58.58 (12)
N2—N3—C9—C10	−176.86 (9)	C15—C14—C18—C10	−59.41 (15)
C8—N1—C9—N3	0.04 (11)	C13—C14—C18—C10	60.67 (14)
C7—N1—C9—N3	−179.32 (10)	C9—C10—C18—C14	−178.04 (10)
C8—N1—C9—C10	175.88 (9)	C11—C10—C18—C14	−59.25 (14)
C7—N1—C9—C10	−3.48 (16)	C17—C10—C18—C14	58.16 (13)
N3—C9—C10—C11	−13.20 (14)	C13—C12—C19—C16	60.51 (14)
N1—C9—C10—C11	171.41 (10)	C11—C12—C19—C16	−59.68 (15)
N3—C9—C10—C18	105.64 (11)	C15—C16—C19—C12	−60.60 (14)
N1—C9—C10—C18	−69.75 (12)	C17—C16—C19—C12	60.06 (14)

### Hydrogen-bond geometry (Å, º)

Cg1 is the centroid of the N1–N3/C8/C9 triazole ring.

**Table:**

*D*—H···*A*	*D*—H	H···*A*	*D*···*A*	*D*—H···*A*
N2—H1*N*2···S1^i^	0.85 (2)	2.44 (2)	3.2753 (11)	169.1 (18)
C19—H19*B*···*Cg*1^ii^	0.97	2.85	3.7885 (17)	141

Symmetry codes: (i) −*x*+2, −*y*+1, −*z*+1; (ii) *x*−1, *y*, *z*.
